# Structural Evolution, Mechanical Properties, and Thermal Stability of Multi-Principal TiZrHf(Ta, Y, Cr) Alloy Films

**DOI:** 10.3390/ma18153672

**Published:** 2025-08-05

**Authors:** Yung-I Chen, Tzu-Yu Ou, Li-Chun Chang, Yan-Zhi Liao

**Affiliations:** 1 Department of Optoelectronics and Materials Technology, National Taiwan Ocean University, Keelung 202301, Taiwan; outzuyu23@gmail.com (T.-Y.O.); 11289034@mail.ntou.edu.tw (Y.-Z.L.); 2 Department of Materials Engineering, Ming Chi University of Technology, New Taipei 243303, Taiwan; lcchang@mail.mcut.edu.tw; 3 Center for Plasma and Thin Film Technologies, Ming Chi University of Technology, New Taipei 243303, Taiwan

**Keywords:** co-sputtering, crystal structure, mechanical properties, multi-principal element alloys, thermal stability

## Abstract

Mixing enthalpy (Δ*H_mix_*), mixing entropy (Δ*S_mix_*), atomic-size difference (δ), and valence electron concentration (VEC) are the indicators determining the phase structures of multi-principal element alloys. Exploring the relationships between the structures and properties of multi-principal element films is a fundamental study. TiZrHf films with a Δ*H_mix_* of 0.00 kJ/mol, Δ*S_mix_* of 9.11 J/mol·K (1.10R), *δ* of 3.79%, and VEC of 4.00 formed a hexagonal close-packed (HCP) solid solution. Exploring the characterization of TiZrHf films after solving Ta, Y, and Cr atoms with distinct atomic radii is crucial for realizing multi-principal element alloys. This study fabricated TiZrHf, TiZrHfTa, TiZrHfY, and TiZrHfCr films through co-sputtering. The results indicated that TiZrHfTa films formed a single body-centered cubic (BCC) solid solution. In contrast, TiZrHfY films formed a single HCP solid solution, and TiZrHfCr films formed a nanocrystalline BCC solid solution. The crystallization of TiZrHf(Ta, Y, Cr) films and the four indicators mentioned above for multi-principal element alloy structures were correlated. The mechanical properties and thermal stability of the TiZrHf(Ta, Y, Cr) films were investigated.

## 1. Introduction

High-entropy alloys (HEAs) and medium-entropy alloys (MEAs) with multiple principal elements have attracted researchers’ attention due to their specific effects, namely high entropy, lattice distortion, sluggish diffusion, and cocktail effects, which differ from those of traditional alloys designed with the primary constitution and some additives [1]. With these specific effects, HEAs and MEAs have been developed in versatile applications, such as alloys with high strength and ductility at cryogenic temperatures [2], hydrogen storage materials [3], diffusion barriers [4,5,6], and biocompatible implants [7,8]. The mixing enthalpy (Δ*H*_mix_), mixing entropy (Δ*S*_mix_), and atomic-size difference (δ) of multi-component alloys were vital indicators that influenced the phase structures of HEAs and MEAs [9]. For example, the alloys with low δ, high Δ*S*_mix_, and not very negative Δ*H*_mix_ tended to form a compound with a single solid solution crystallizing into a simple structure. Moreover, alloys’ valence electron concentration (VEC) determines the solid solutions’ phase structures [10,11]. The alloys exhibited hexagonal close-packed (hcp), body-centered cubic (bcc), and face-centered cubic (fcc) phases as their VEC were <4.09 [11], 4.18–6.87 [10,11], and ≥8 [10], respectively. Ti, Zr, and Hf, with a VEC of 4, can crystallize into either hcp or bcc structures [12]. In a previous study [13], TiNbTa(Zr) films with a VEC value of 4.40–4.90 exhibited a bcc phase, whereas the other TiNbTa(Zr) films with VEC values of 4.17–4.24 showed a hcp and bcc mixed phase. Ta and Nb are biocompatible and known as bcc-phase stabilizers in Ti-based alloys [14], and their oxide, Ta_2_O_5_ and Nb_2_O_5,_ are recognized as passivation films during corrosion tests [15]. RHEAs primarily comprise refractory metal elements from Ti, Zr, Hf, V, Nb, Ta, Cr, Mo, and W. These alloys have gathered widespread attention due to their suitability for high-temperature applications [16]. According to the Hume-Rothery criteria established for binary alloy systems, a solid solution forms when the solute and solvent elements have an atomic size difference of less than 15%. However, a critical indicator δ of 0.065 distinguished solid solution formation and amorphous structures for HEA systems [17]. Moreover, a more negative Δ*H*_mix_ favors the formation of an amorphous phase [17]. Ta, with a VEC of 5, has an atomic radius of 0.143 nm, which is slightly lower than those of Ti (0.14615 nm), Zr (0.16025 nm), and Hf (0.15775 nm) [18]. In contrast, Y, with a low VEC value of 3, possesses a large atomic radius of 0.18015 nm, whereas Cr, with a high VEC of 6, has a small atomic radius of 0.12491 nm [18]. Investigating the phase structures and characteristics of TiZrHfM films with distinct metallic atoms (M = Ta, Y, Cr) becomes the topic of this study. Moreover, the thermal stability of these phases through vacuum annealing in the temperature range of 500–800 °C was investigated.

## 2. Materials and Methods

**Figure 1:**
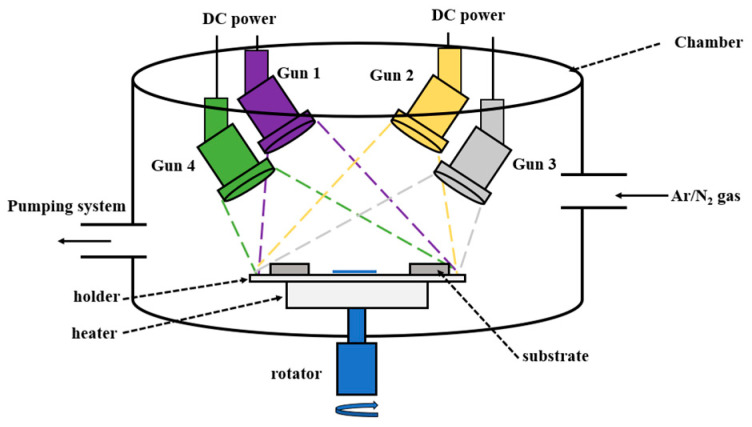
Schematic of the co-sputtering apparatus.

TiZrHfM (M = Ta, Y, Cr) films were prepared through a four-gun co-sputtering system, as illustrated in Figure 1. Direct current (DC) powers applied to the sputter guns are listed in Table 1. Targets of Ti (99.995%), Zr (99.9%), Hf (99.99%), Ta (99.99%), Y (99.99%), and Cr (99.99%) (Ultimate Materials Technology, Jubei, Taiwan) with a diameter and thickness of 50.8 and 6 nm, respectively, were adopted. A Ti interlayer was deposited on the substrates using a DC power of 200 W, an Ar flow rate of 20 sccm, and a substrate holder rotation speed of 30 rpm, which formed a thickness of 90–181 nm. Then, TiZrHf, TiZrHfTa, TiZrHfY, and TiZrHfCr films of 685–1272 nm were deposited on the Ti interlayer. Vacuum annealing was performed in 5.3 × 10^−3^ Pa at 500–800 °C for 1 h. The heating rate was 20 °C/min, and the annealed sample was furnace cooled to <50 °C in 2 h. Film thicknesses and surface morphologies were examined using a field emission scanning electron microscope (JSM-IT700HR, JEOL, Tokyo, Japan). The chemical compositions of the films were determined using a field-emission electron probe microanalyzer (JXA-iHP200F, JEOL, Tokyo, Japan). Three measurements for each sample were used for calculating the standard deviations of thickness and compositions. Phase identification was conducted using grazing incident X-ray diffraction (GIXRD; X’Pert PRO MPD, PANalytical, Almelo, The Netherlands). Lattice constants (*ɑ*_0_) of the films were decided from GIXRD reflections by using the equation [19]:

(1) $$a=a_{0}+K\times \frac{\cos^{2}\theta }{\sin\theta }$$

where *ɑ* is the lattice constant for a distinct reflection, *K* is a constant, and *θ* is the diffraction angle. Although standard deviations are not provided, the uncertainty in lattice constant estimation is primarily influenced by instrumental resolution, peak fitting accuracy, and alignment. Based on typical GIXRD setups and analysis methods, the relative uncertainty is estimated to be within ±0.5%. The hardness and elastic modulus of films were measured using a nanoindentation tester (TI-900 Triboindenter, Hysitron, Minneapolis, MN, USA) equipped with a Berkovich diamond probe tip, and the data calculations were performed using the Oliver–Pharr method [20]. The indentation depth was controlled at 80 nm to minimize substrate influence. Six measurements for each sample were used to determine the standard deviation. Residual stress in the films was determined using the curvature method [21]. Corrosion experiments of the films prepared on SUS420 substrates were carried out in a 3.5 wt.% NaCl aqueous solution with a pH value of 6.3 using a potentiodynamic polarization test setup (SP-200, BioLogic, Seyssinet-Pariset, France) with an Ag/AgCl reference electrode and a Pt counter electrode. The samples with C and Pt protective layers for transmission electron microscopy (TEM, JEM-2010F, JEOL, Tokyo, Japan) observation were prepared using a focused ion beam system (NX2000, Hitachi, Tokyo, Japan).

## 3. Results and Discussion

### 3.1. Chemical Compositions and Phases

**Figure 2:**
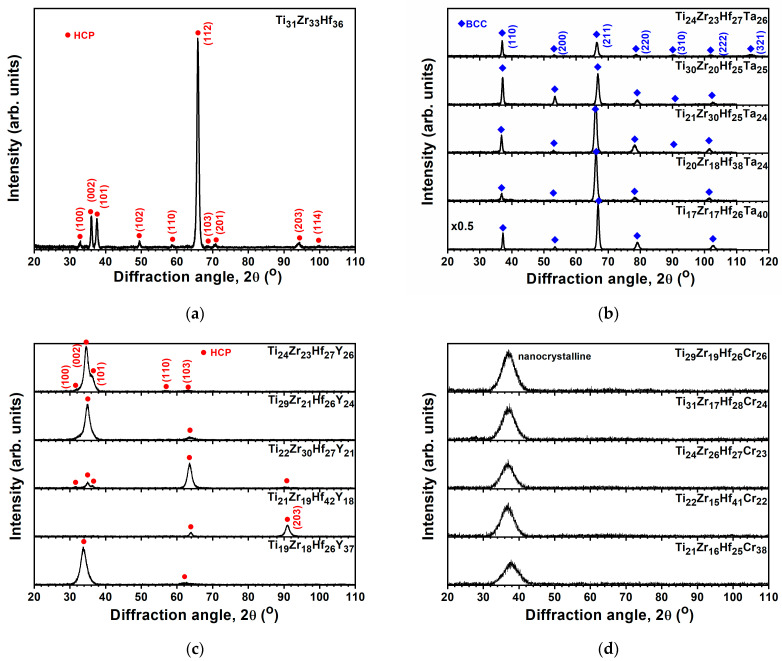
GIXRD patterns of the (**a**) TiZrHf, (**b**) TiZrHfTa, (**c**) TiZrHfY, and (**d**) TiZrHfCr films.

Table 2 lists the chemical compositions of the TiZrHf(Ta, Y, Cr) films. The O contents in the TiZrHf, TiZrHfTa, and TiZrHfY films were in the range of 3.4–6.5 at.%, whereas the TiZrHfCr films had a lower O content of 0–2.6 at.%. These TiZrHf(Ta, Y, Cr) films were denoted by their metallic compositions. The calculated VEC, δ, Δ*H*_mix_, and Δ*S*_mix_ values, without considering the oxygen content, are shown in Table 2. The Δ*S*_mix_ of Ti_31_Zr_33_Hf_36_ film was 9.11 J/mol K or 1.10 R, whereas the Δ*S*_mix_ values of all the prepared TiZrHf(Ta, Y, Cr) films were in the range of 1.31–1.38 R, classified as MEAs. The equilibrium state of Ti, Zr, and Hf at room temperature is a close-packed hexagonal (HCP) structure (ICDD 00-044-1294, 00-005-0665, and 00-038-1478). It is expected that the TiZrHf alloys should exhibit a single HCP structure [22]. However, Tsai et al. [23] reported a TiZrHf film exhibiting a body-centered cubic (BCC) structure. This discrepancy may arise due to the non-equilibrium nature of the sputtering process involving high-energy particle collisions, leading to the formation of high-temperature phases for the TiZrHf films. In this study, a co-sputtered Ti_31_Zr_33_Hf_36_ film exhibited an HCP phase, as shown in Figure 2a. Figure 2b displays that all the surveyed TiZrHfTa films exhibited a single BCC phase, either for the near-equiatomic Ti_24_Zr_23_Hf_27_Ta_26_ or Ti-enriched Ti_30_Zr_20_Hf_25_Ta_25_, Zr-enriched Ti_21_Zr_30_Hf_25_Ta_24_, Hf-enriched Ti_20_Zr_18_Hf_38_Ta_24_, and Ta-enriched Ti_17_Zr_17_Hf_26_Ta_40_ films. The presence of (321) reflection, the seventh one, of the Ti_24_Zr_23_Hf_27_Ta_26_ film indicates that this phase is a BCC phase, not a simple cubic phase. The lattice constants of Ti_24_Zr_23_Hf_27_Ta_26_, Ti_30_Zr_20_Hf_25_Ta_25_, Ti_21_Zr_30_Hf_25_Ta_24_, Ti_20_Zr_18_Hf_38_Ta_24_, and Ti_17_Zr_17_Hf_26_Ta_40_ films were determined to be 0.3438, 0.3426, 0.3449, 0.3452, and 0.3426 nm, respectively, which interlaid those of BCC Ti (0.33065 nm, ICDD 00-044-1288), Zr (0.35453 nm, ICDD 00-034-06570), Hf (0.35 nm, ICDD 01-089-5154), and Ta (0.33058 nm, ICDD 00-004-0788). The high-entropy and rapid quenching effects resulted in the high-entropy alloys (HEAs) forming single-phase structures rather than complex intermetallic compounds. The δ values were 3.79% for the Ti_31_Zr_33_Hf_36_ film and 4.54–4.82% for the TiZrHfTa films, which implied the formation of solid solutions as δ < 6.5% [17]. Moreover, the VEC value was 4.00 for the Ti_31_Zr_33_Hf_36_ film and 4.24–4.40 for the TiZrHfTa films, indicating their solid solutions were HCP and BCC phases [11], respectively. In contrast, the GIXRD patterns of TiZrHfY films exhibited an HCP phase (Figure 2c), whereas the TiZrHfCr films seemed nanocrystalline (Figure 2d). The δ, Δ*H*_mix_, and VEC values were 6.81–8.07%, 6.89–10.91 kJ/mol, and 3.63–3.82 for the TiZrHfY films, respectively, whose structures could not be identified according to the δ-Δ*H*_mix_ phase selection rule proposed in [17] ascribed to limited data. Braic et al. [22] reported that the TiZrHfY film with a δ of 7.6% had two HCP phases, whereas the TiZrY film with a high δ of 8.6% was amorphous. Ti, Zr, Hf, and Y tend to form HCP phases. The large Y atom has a 16% expansion related to the average atom size of Ti, Zr, and Hf, which enlarges the lattice constants of an HCP structure for TiZrHfY films. By contrast, the small Cr atom reveals a −19% shrinkage related to the average atom size of Ti, Zr, and Hf, which makes the lattice unstable and reduces long-range order crystallinity. Therefore, the fabricated TiZrHfCr films were nanocrystalline or X-ray amorphous. The δ, Δ*H*_mix_, and VEC values were 8.87–10.41%, 6.19–8.61 kJ/mol, and 4.44–4.77 for the TiZrHfCr films in this study. δ plays a critical role in forming amorphous phases [24]. Regarding kinetics, magnetron sputtering technology’s lattice distortion and rapid quenching effects alleviate the diffusion of deposited atoms, promoting the emergence of nanocrystalline and amorphous composite structures in thin films.

**Figure 3:**
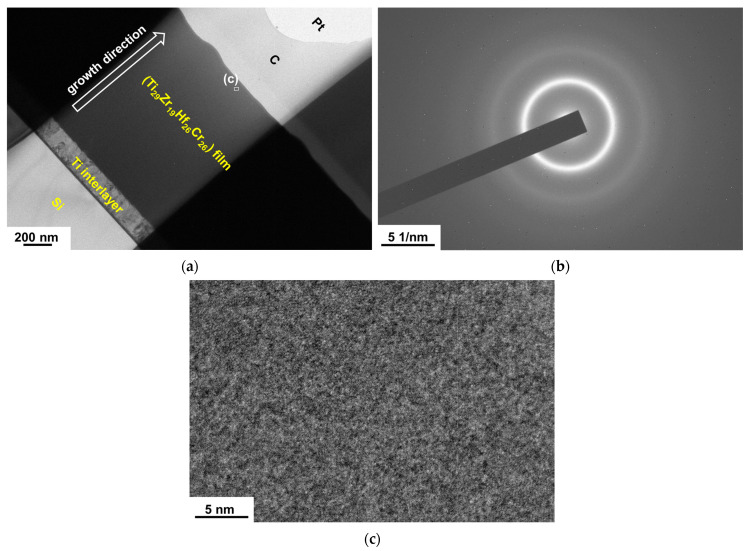
(**a**) XTEM, (**b**) SAED, and (**c**) HRTEM images of the Ti_29_Zr_19_Hf_26_Cr_26_ film.

Our previous studies have reported the TEM observations of BCC TiZrHfTa [25] and HCP TiZrHfY [26] films, which exhibit coarse columnar structures. Figure 3a depicts the cross-sectional TEM (XTEM) image of the Ti_29_Zr_19_Hf_26_Cr_26_ film, distinguishing the Ti interlayer and the Ti_29_Zr_19_Hf_26_Cr_26_ film. The Ti_29_Zr_19_Hf_26_Cr_26_ film exhibits a featureless morphology, indicating the formation of an almost amorphous or nanocrystalline structure. The selected area electron diffraction (SAED) pattern reveals diffused rings, implying the formation of a nanocrystalline structure (Figure 3b). Figure 3c shows a high-resolution TEM (HRTEM) image with no distinct crystalline regions in which no well-defined crystalline domains are observed, further indicating that the crystallites are extremely fine. Structural characterization, including GIXRD and HRTEM analysis, confirms that the Ti_29_Zr_19_Hf_26_Cr_26_ thin film possesses a nanocrystalline structure.

### 3.2. Mechanical and Anticorrosive Properties of TiZrH(Ta, Y, Cr) Films

Table 3 lists the hardness (*H*) and elastic modulus (*E*) values of TiZrHf, TiZrHfTa, TiZrHfY, and TiZrHfCr films. The Ti_31_Zr_33_Hf_36_ film had *H* and *E* values of 7.4 and 179 GPa, respectively, an elastic recovery (*W_e_*) of 31%, and a residual stress of −0.13 GPa. The TiZrHfTa films had *H* and *E* values of 4.6–5.4 and 100–114 GPa, respectively, lower than those of the Ti_31_Zr_33_Hf_36_ film. The TiZrHfTa films had *W_e_* values of 28–33% and residual stresses ranging from 0 to −0.23 GPa, comparable to those of the Ti_31_Zr_33_Hf_36_ film. The TiZrHfY films had *H* and *E* values of 6.0–7.9 and 106–134 GPa, respectively. The *H* values of the TiZrHfY films were comparable to those of the Ti_31_Zr_33_Hf_36_ film, accompanied by higher *W_e_* values of 33–38% and larger compressive residual stresses between −0.41 and −0.74 GPa. However, the *E* values of the TiZrHfY films were at low levels of 106–134 GPa. The TiZrHfCr films had *H* and *E* values of 5.3–8.4 and 91–113 GPa, respectively, *W_e_* values of 43–48%, and residual stresses ranging from −0.15 to −0.43 GPa. The TiZrHfY film exhibited enhanced mechanical performance and higher compressive residual stress, primarily attributed to Y’s relatively large atomic radius. Incorporating Y atoms increases the lattice parameters of the HCP structure, thereby intensifying lattice mismatch and structural stress. Additionally, the significant atomic size difference between Y and the other constituent elements promotes solid solution strengthening, further contributing to the improved mechanical properties of the TiZrHfY film.

**Figure 4:**
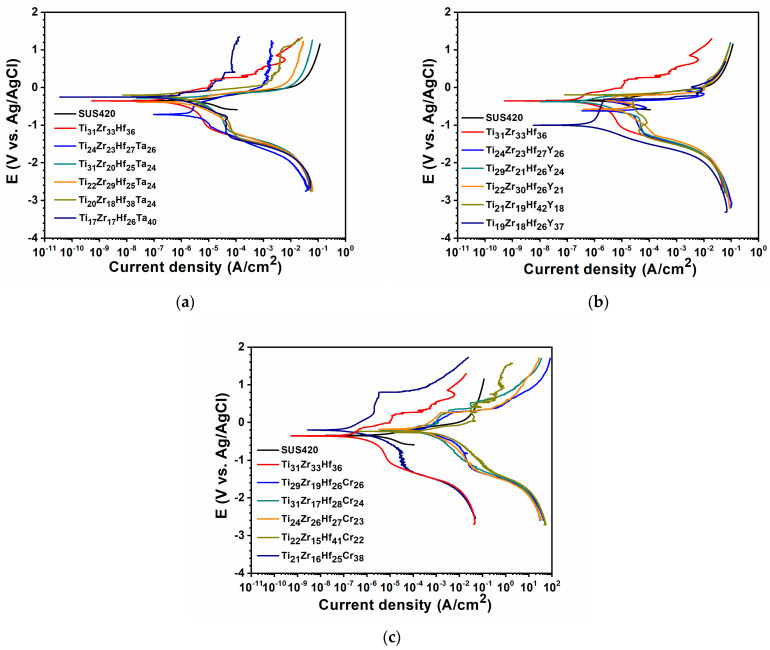
Potentiodynamic polarization curves of SUS420 substrate, Ti_31_Zr_33_Hf_36_ film, and (**a**) TiZrHfTa, (**b**) TiZrHfY, and (**c**) TiZrHfCr films tested in a 3.5 wt% NaCl aqueous solution.

Figure 4 depicts the potentiodynamic polarization curves of SUS420 substrate, Ti_31_Zr_33_Hf_36_, TiZrHfTa, TiZrHfY, and TiZrHfCr films tested in a 3.5 wt.% NaCl aqueous solution and the relevant data are summarized in Table 4. While only one specimen was tested per film, possible uncertainties in corrosion parameters may result from variations in surface condition, measurement reproducibility, and reference electrode stability. Based on experience with similar tests, the accuracy of polarization resistance (*R_p_*) values is estimated to be within ±10%. The Ti_31_Zr_33_Hf_36_ film exhibited an *I_corr_* value of 0.338 μA/cm^2^ and an *E_corr_* value of −354 mV, along with a high polarization resistance *R_p_* of 1.9 × 10^5^ Ω·cm^2^, indicating excellent corrosion resistance. Numerous studies have reported that refractory metals in TiZr-based refractory high-entropy alloys tend to form dense and highly protective oxide layers on the surface, thereby imparting superior corrosion resistance [27,28,29,30]. Compared to the Ti_31_Zr_33_Hf_36_ film, adding Ta did not enhance the corrosion resistance. In contrast, the film with Y addition exhibited the poorest corrosion resistance, likely due to the formation of Y oxides that provide active pathways for corrosive species, thereby facilitating penetration and compromising the film’s protective capability [31,32]. The TiZrHfCr film demonstrated significantly improved corrosion resistance, as Cr is known to be one of the most effective elements for enhancing the corrosion resistance of coatings. The incorporation of an appropriate amount of Cr promotes the formation of a continuous and uniform Cr_2_O_3_ passivation layer on the surface, which effectively protects the coating from corrosion [33,34].

### 3.3. Thermal Stability of TiZrHf(Ta, Y, Cr) Films

**Figure 5:**
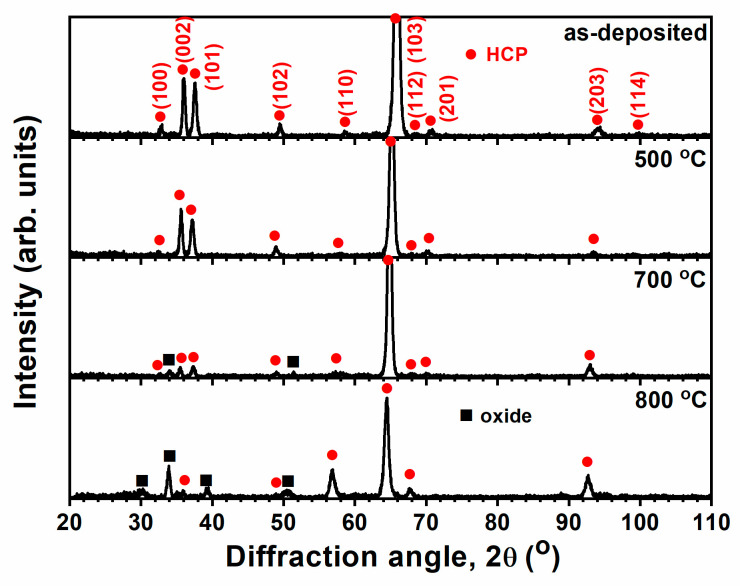
GIXRD patterns of the Ti_31_Zr_33_Hf_36_ films annealed at 500, 700, and 800 °C for 1 h.

**Figure 6:**
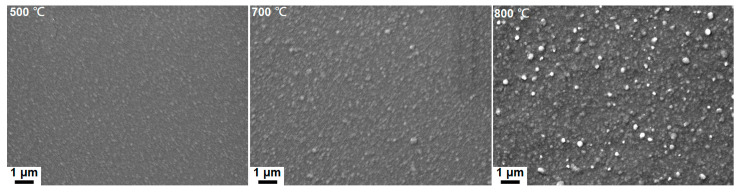
Surface morphologies of the Ti_31_Zr_33_Hf_36_ films annealed at 500, 700, and 800 °C for 1 h.

The thermal stability of TiZrHf(Ta, Y, Cr) films was evaluated by annealing these films in a vacuum tube furnace. Figure 5 displays the GIXRD patterns of the Ti_31_Zr_33_Hf_36_ films annealed at 500, 700, and 800 °C. The structure of the Ti_31_Zr_33_Hf_36_ film annealed at 500 °C maintained an HCP phase, whereas the Ti_31_Zr_33_Hf_36_ films annealed at 700 and 800 °C showed an additional oxide phase (ICDD 00-042-1164). Figure 6 depicts the surface morphologies of the annealed Ti_31_Zr_33_Hf_36_ films. Granular oxide particles were observed on the surfaces of 700 and 800 °C-annealed Ti_31_Zr_33_Hf_36_ films. After annealing at high temperatures, the phase splitting for multi-principal element alloys was not observed for the Ti_31_Zr_33_Hf_36_ films. The hardness value of the Ti_31_Zr_33_Hf_36_ film was 7.4 GPa at the as-deposited state and was increased to 11.6 and 18.8 GPa, and then decreased to 17.6 GPa as the film was annealed at 500, 700, and 800 °C, respectively. The elastic modulus value was 179 GPa at the as-deposited state and was varied to 171, 241, and 226 GPa as the film was annealed at 500, 700, and 800 °C, respectively. The oxide phase formation and randomly disposed geometry increased the mechanical properties of the Ti_31_Zr_33_Hf_36_ films.

**Figure 7:**
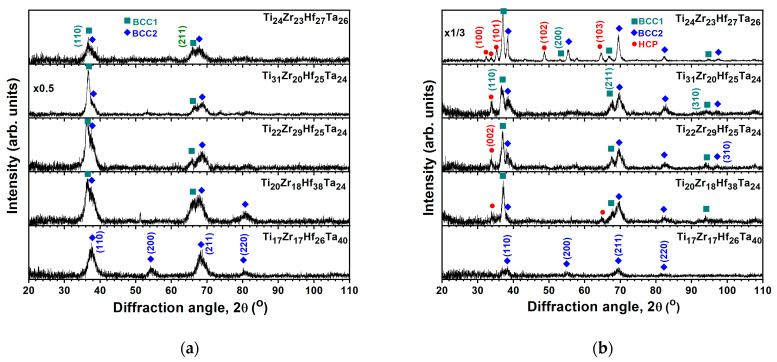
GIXRD patterns of the TiZrHfTa films annealed for 1 h at (**a**) 500 and (**b**) 700 °C.

**Figure 8:**
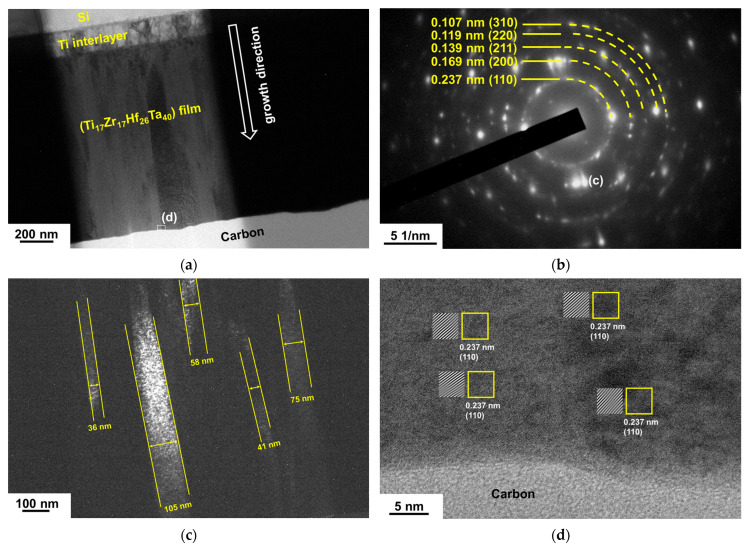
(**a**) XTEM, (**b**) SAED, (**c**) dark-field, and (**d**) HRTEM images of the Ti_17_Zr_17_Hf_26_Ta_40_ film after annealing at 500 °C for 1 h.

**Figure 9:**
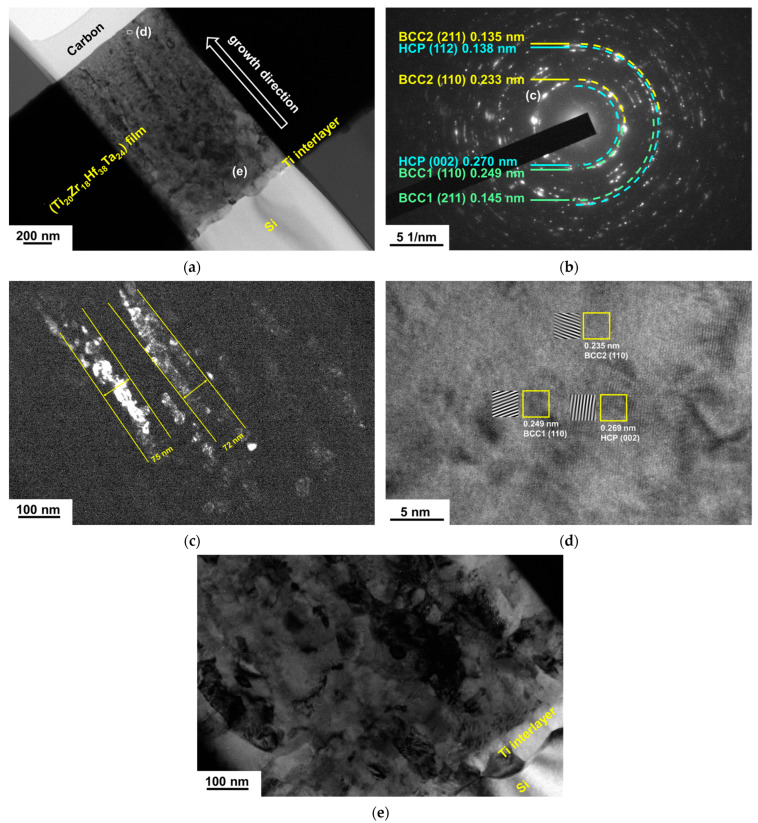
(**a**) XTEM image of the Ti_20_Zr_18_Hf_38_Ta_24_ film after annealing at 700 °C for 1 h; (**b**) SAED, (**c**) dark-field, and (**d**) HRTEM images of the outer part of the film; (**e**) XTEM image of the inner part of the film.

Figure 7a depicts the GIXRD patterns of the TiZrHfTa films annealed in a vacuum at 500 °C for 1 h. The Ti_17_Zr_17_Hf_26_Ta_40_ film maintained a BCC phase, namely BCC2; however, the peak widths broadened, implying recrystallization of the film. Figure 8a depicts an XTEM image of the 500 °C-annealed Ti_17_Zr_17_Hf_26_Ta_40_ film. The SAED pattern reveals a BCC structure with (110), (200), (211), (220), and (310) diffraction rings (Figure 8b). Figure 8c displays the dark-field image related to the (200) diffraction spot shown in Figure 8b, which exhibits a columnar structure with 36–105 nm widths. The HRTEM image shown in Figure 8d reveals lattice fringes with a *d*-spacing of 0.237 nm correlated to the BCC2 (110) planes. In contrast, all the other TiZrHfTa films exhibited phase separation after annealing at 500 °C. The as-deposited BCC phase split into two BCC phases (Figure 7a); the new BCC phase, namely BCC1, had *d*-spacings larger than those of BCC2. Moreover, an HCP phase appeared after these TiZrHfTa films were annealed at 700 °C (Figure 7b). Yao et al. [35] and Chen et al. [36] reported that bulk metallic HfNbTaTiZr alloys transformed into a BCC Ta–Nb, an HCP Hf–Zr, and a BCC matrix after annealing at 500–700 °C and 550–700 °C, respectively. The phase separation due to annealing did not occur at 500 and 700 °C for the Ti_17_Zr_17_Hf_26_Ta_40_ film. Cheng et al. [37] reported that (TiZrHf)*_x_*(NbTa)_1−*x*_ thin films with 0.55 < *x* < 0.7 exhibited high resistance to crystallization up to 500 °C. Figure 9a presents an XTEM image of the Ti_20_Zr_18_Hf_38_Ta_24_ film after annealing at 700 °C for 1 h. The SAED image from the outer part of the film reveals two BCC phases, BCC1 and BCC2, and an HCP phase (Figure 9b). Figure 9c displays a dark-field image related to the BCC2 (110) diffraction spot shown in Figure 9b. The outer part of the annealed films maintained a columnar structure with widths of 72–75 nm. Figure 9d shows an HRTEM image, which reveals lattice fringes with *d*-spacings of 0.249, 0.235, and 0.268 nm correlating to BCC1 (110), BCC2 (110), and HCP (002) planes, respectively. By contrast, the inner part of the film adjacent to the Ti interlayer consists of a granular structure, as shown in Figure 9e. According to the structure-zone model for sputtered films [38], a columnar structure is familiar. Annealing provides thermal energy that accelerates atomic diffusion. As sputtered films are annealed, energetic atoms could move along grain boundaries, promote grain growth and defect annihilation, and form a coarser columnar structure, accompanied by well-aligned crystallographic orientation. In contrast, recrystallization and grain growth at high temperatures tend to create a granular structure attributed to the driving force of reducing free energy. The hardness values of the TiZrHfTa films increased from 4.6 to 5.4 GPa at the as-deposited state to 6.8–7.9 and 11.2–12.5 GPa after they were annealed at 500 and 700 °C, respectively, as shown in Table 5. The elastic moduli increased from 100–114 GPa at the as-deposited state to 128–144 and 182–196 GPa after 500 and 700 °C annealing, respectively.

**Figure 10:**
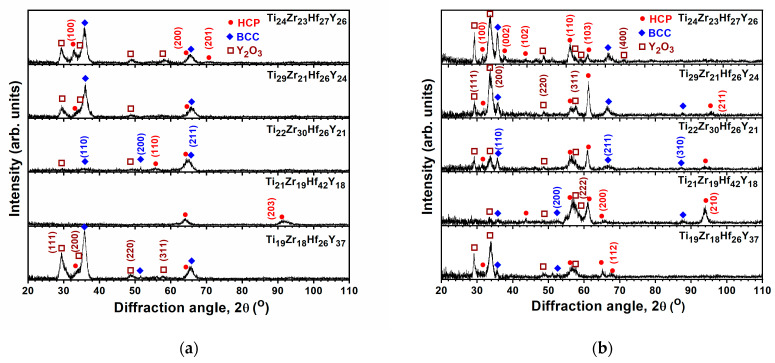
GIXRD patterns of the TiZrHfY films annealed at (**a**) 500 and (**b**) 700 °C for 1 h.

**Figure 11:**
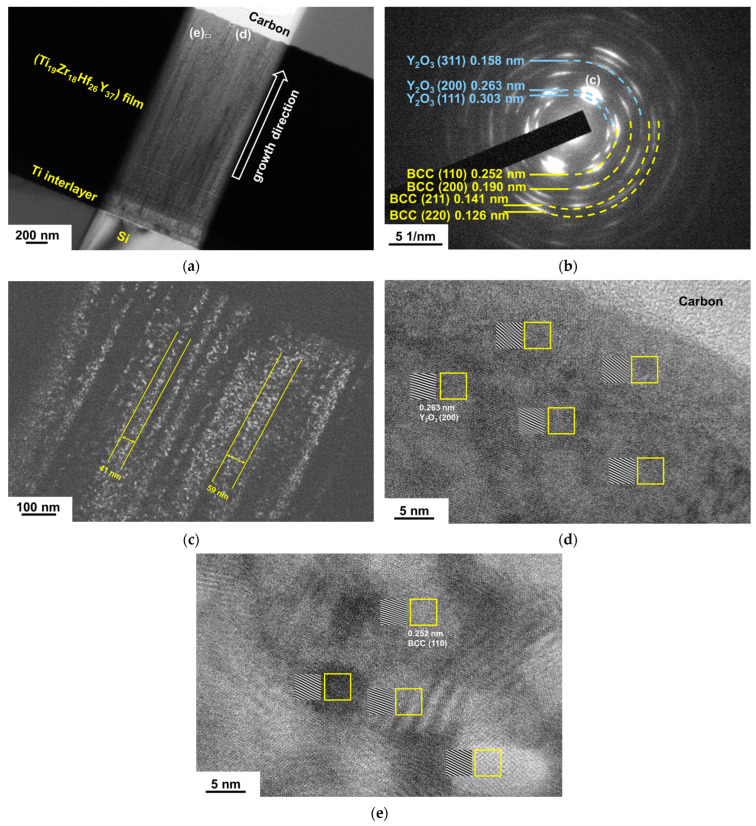
(**a**) XTEM, (**b**) SAED, and (**c**) dark-field images of the Ti_19_Zr_18_Hf_26_Y_37_ film after annealing at 500 °C for 1 h; HRTEM images at the near-surface region (**d**) and the inner part (**e**) of the film.

Figure 10 depicts the GIXRD patterns of TiZrHfY thin films after they were annealed at 500 and 700 °C for 1 h. The as-deposited HCP phase of most of the TiZrHfY films splits into a combination of HCP and BCC phases, along with the formation of a cubic Y_2_O_3_ oxide [ICDD 00-043-0661] after 500 °C annealing. The Ti_21_Zr_19_Hf_42_Y_18_ film with high Hf and low Y contents maintained the original HCP phase. All the TiZrHfY films exhibited similar XRD reflections after 700 °C annealing. The standard Gibbs free energies of oxide formation at 500 °C are −1119, −803, −950, and −1001 kJ/(mol of O_2_) for Y_2_O_3_, TiO_2_, ZrO_2_, and HfO_2_ [39], respectively. Y_2_O_3_ is the dominant oxide phase. Figure 11a displays an XTEM image of the Ti_19_Zr_18_Hf_26_Y_37_ after annealing at 500 °C for 1 h. Figure 11b shows an SAED pattern with BCC (110), (200), (211), and (220) and Y_2_O_3_ (111), (200), and (311) signals. The diffraction ring for HCP (100) plans was insignificant, and it lay between Y_2_O_3_ (111) and (200) signals. Figure 11c shows a dark-field image correlated to the BCC (110) diffraction point in the SAED pattern, which displays a columnar structure with widths of 41–59 nm. Figure 11d exhibits an HRTEM image near the sample’s surface, which reveals lattice fringes with a *d*-spacing of 0.263 nm belonging to Y_2_O_3_ (200) planes. Figure 11e presents an HRTEM image for the region beneath the sample’s surface, as Figure 11a shows, which displays lattice fringes with a *d*-spacing of 0.252 nm for BCC (110) planes. The hardnesses of the TiZrHfY films increased from 6.0–7.9 GPa at the as-deposited state to 8.4–11.6 and 8.5–15.8 GPa (Table 5) after they were annealed at 500 and 700 °C, respectively. The elastic moduli increased from 104–134 GPa at the as-deposited state to 132–199 and 170–236 GPa after 500 and 700 °C annealing, respectively. The increases in mechanical properties after annealing for the TiZrHfY films were not as significant as those of the annealed TiZrHfTa and TiZrHfCr films. This may be attributed to the influence of Y_2_O_3_ oxides, which possess a low hardness in the range of 8–9 GPa [40]. Xu et al. [41] reported that cubic Y_2_O_3_ films exhibited an optimal hardness of 12 GPa after 350 °C annealing, whereas the hardness decreased to 7–9 GPa after 500–650 °C annealing. The improvement on mechanical properties of the annealed TiZrHfY films contributed from oxides was limited.

**Figure 12:**
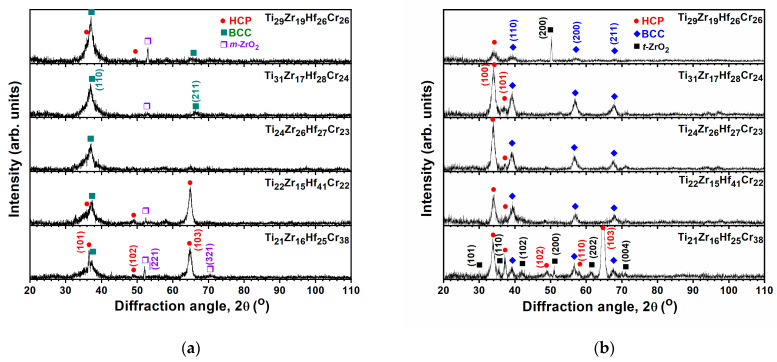
GIXRD patterns of the TiZrHfCr films annealed at (**a**) 500 and (**b**) 700 °C for 1 h.

Figure 12 displays GIXRD patterns of the TiZrHfCr thin films annealed at 500 and 700 °C for 1 h. BCC and HCP phases were observed, accompanied by the appearance of ZrO_2_ oxide phases. The ZrO_2_ phases were monoclinic (ICDD 00-037-1484) and tetragonal (ICDD 00-042-1164) at 500 and 700 °C, respectively. The hardnesses of the TiZrHfCr films increased from 5.3–8.4 GPa at the as-deposited state to 9.0–11.6 and 15.0–20.6 GPa (Table 5) after they were annealed at 500 and 700 °C, respectively. The elastic moduli increased from 91–113 GPa at the as-deposited state to 139–154 and 202–241 GPa after 500 and 700 °C annealing, respectively. The mechanical properties of the 700 °C-annealed TiZrHfCr films were the highest values within the studied TiZrHf(Ta, Y, Cr) films. This may be attributed to the structural transformation of the TiZrHfCr film from an amorphous phase in the as-deposited state to a mixed BCC and HCP phase after 1 h of annealing. Moreover, hardness values of 12–19 GPa were reported for ZrO_2_ thin films [42,43,44], which implied the oxide phase could contribute to the enhancement of mechanical properties for the annealed TiZrHfCr films.

## 4. Conclusions

In this study, a series of TiZrHf-based multi-principal element alloy thin films were successfully fabricated, and the effects of incorporating Ta, Y, and Cr on their phase structure, mechanical properties, corrosion resistance, and thermal stability were systematically investigated. The TiZrHf film, with appropriate mixing entropy, atomic size mismatch, and valence electron concentration, formed a hexagonal close-packed solid solution, representing a typical medium-entropy alloy thin film system. Adding Ta stabilized the formation of a single-phase body-centered cubic structure, although no significant improvement in corrosion resistance was observed. In contrast, incorporating Y resulted in the poorest corrosion performance, likely due to the formation of porous Y_2_O_3_ oxides that act as active pathways for corrosive agents. On the other hand, the Cr addition markedly enhanced both corrosion resistance—through forming a dense and continuous Cr_2_O_3_ passivation layer—and mechanical strength. The Ti_31_Zr_17_Hf_28_Cr_24_ film exhibited an *I_corr_* value of 0.064 μA/cm^2^ and an *E_corr_* value of −202 mV, along with the highest *R_p_* (4.5 × 10^5^ Ω·cm^2^), which was 58.1 and 2.3 times higher than that of the SUS420 substrate and Ti_31_Zr_33_Hf_36_ film, respectively. Regarding thermal stability, the TiZrHfCr film exhibited the highest hardness and elastic modulus even after annealing at elevated temperatures up to 700 °C. It indicates superior structural integrity and mechanical performance, making it a promising candidate for high-temperature protective coatings. Overall, the results demonstrate that compositional engineering is a practical approach to tailoring the phase formation and functional properties of TiZrHf-based thin films, providing a fundamental basis for developing advanced multi-principal element alloy coatings.

## Figures and Tables

**Table 1:** Target, power arrangements, and thickness values for co-sputtering TiZrHf (Ta, Y, Cr) films.

Sample	Gun 1	Gun 2	Gun 3	Gun 4	*T_F_* ^1^ (nm)	*T_I_* ^2^ (nm)
	*P*_Ti_ ^3^	*P*_Hf_	*P*_Zr_			
Ti_31_Zr_33_Hf_36_	120	80	100		796 ± 9	131 ± 0
	*P*_Ti_	*P*_Hf_	*P*_Zr_	*P*_Ta_		
Ti_24_Zr_23_Hf_27_Ta_26_	120	80	120	80	916 ± 8	143 ± 3
Ti_30_Zr_20_Hf_25_Ta_25_	170	80	120	80	1058 ± 10	135 ± 1
Ti_21_Zr_30_Hf_25_Ta_24_	120	80	170	80	795 ± 8	181 ± 8
Ti_20_Zr_18_Hf_38_Ta_24_	120	130	120	80	923 ± 13	163 ± 3
Ti_17_Zr_17_Hf_26_Ta_40_	120	80	120	130	685 ± 5	102 ± 8
	*P*_Ti_	*P*_Hf_	*P*_Zr_	*P*_Y_		
Ti_24_Zr_23_Hf_27_Y_26_	130	80	90	100	1046 ± 7	117 ± 1
Ti_29_Zr_21_Hf_26_Y_24_	180	80	90	100	1110 ± 10	117 ± 6
Ti_22_Zr_30_Hf_27_Y_21_	130	80	140	100	1145 ± 3	115 ± 7
Ti_21_Zr_19_Hf_42_Y_18_	130	130	90	100	1189 ± 6	117 ± 6
Ti_19_Zr_18_Hf_26_Y_37_	130	80	90	150	1272 ± 2	124 ± 1
	*P*_Ti_	*P*_Hf_	*P*_Zr_	*P*_Cr_		
Ti_29_Zr_19_Hf_26_Cr_26_	150	90	100	60	1133 ± 7	139 ± 9
Ti_31_Zr_17_Hf_28_Cr_24_	200	90	100	60	1129 ± 10	137 ± 0
Ti_24_Zr_26_Hf_27_Cr_23_	150	90	150	60	1119 ± 11	90 ± 10
Ti_22_Zr_15_Hf_41_Cr_22_	150	140	100	60	1165 ± 5	125 ± 2
Ti_21_Zr_16_Hf_25_Cr_38_	150	90	100	110	1200 ± 7	107 ± 0

^1^ *T_F_*: film thickness. ^2^ *T_I_*: interlayer thickness. ^3^ *P*: powers applied on the targets. Power unit: watts.

**Table 2:** Chemical compositions, valence electron concentration, atomic-size difference, mixing enthalpy, and mixing entropy of TiZrHf(Ta, Y, Cr) films.

Sample	Chemical Composition (at.%)					VEC ^1^	δ ^2^	Δ*H*_mix_ ^3^	Δ*S*_mix_ ^4^
	Ti	Zr	Hf	Ta/Y/Cr	O		(%)	(kJ/mol)	(J/mol K)
Ti_31_Zr_33_Hf_36_	28.9 ± 0.4	31.5 ± 0.6	34.5 ± 0.3	-	5.1 ± 0.1	4.00	3.79	0	9.11
Ti_24_Zr_23_Hf_27_Ta_26_	22.7 ± 0.0	21.2 ± 0.4	25.9 ± 0.4	24.9 ± 0.2	5.3 ± 0.4	4.26	4.70	1.82	11.50
Ti_30_Zr_20_Hf_25_Ta_25_	28.7 ± 0.3	19.1 ± 0.2	23.9 ± 0.3	23.1 ± 0.3	5.2 ± 0.4	4.24	4.62	1.62	11.44
Ti_21_Zr_30_Hf_25_Ta_24_	20.2 ± 0.4	27.8 ± 0.2	23.9 ± 0.1	22.5 ± 0.1	5.6 ± 0.3	4.24	4.74	1.77	11.47
Ti_20_Zr_18_Hf_38_Ta_24_	18.6 ± 0.8	16.6 ± 0.5	36.1 ± 0.5	22.7 ± 0.6	6.0 ± 0.4	4.24	4.54	1.82	11.12
Ti_17_Zr_17_Hf_26_Ta_40_	15.7 ± 0.8	16.5 ± 0.4	24.9 ± 0.2	37.7 ± 0.4	5.2 ± 0.5	4.40	4.82	2.35	10.97
Ti_24_Zr_23_Hf_27_Y_26_	22.0 ± 0.4	21.3 ± 0.5	25.4 ± 0.3	24.8 ± 0.7	6.5 ± 1.7	3.73	7.72	9.09	11.50
Ti_29_Zr_21_Hf_26_Y_24_	27.9 ± 0.5	20.0 ± 0.1	24.2 ± 0.2	23.0 ± 0.2	4.9 ± 0.3	3.76	7.85	8.79	11.47
Ti_22_Zr_30_Hf_27_Y_21_	20.4 ± 0.9	28.3 ± 0.5	25.8 ± 0.5	20.0 ± 0.8	5.5 ± 0.2	3.79	7.12	7.57	11.43
Ti_21_Zr_19_Hf_42_Y_18_	19.9 ± 0.4	18.4 ± 0.2	40.7 ± 1.5	17.6 ± 0.9	3.4 ± 3.0	3.82	6.81	6.89	10.94
Ti_19_Zr_18_Hf_26_Y_37_	18.1 ± 0.5	16.5 ± 0.5	24.3 ± 0.0	34.9 ± 0.3	6.2 ± 0.4	3.63	8.07	10.91	10.94
Ti_29_Zr_19_Hf_26_Cr_26_	28.4 ± 0.4	18.2 ±0.6	25.3 ± 0.3	25.7 ± 0.4	2.4 ± 0.5	4.53	9.29	6.96	11.42
Ti_31_Zr_17_Hf_28_Cr_24_	30.2 ± 0.2	17.0 ± 0.3	27.8 ± 0.5	23.3 ± 0.1	1.7 ± 0.3	4.47	8.96	6.42	11.34
Ti_24_Zr_26_Hf_27_Cr_23_	23.6 ± 0.6	25.3 ± 0.4	26.3 ± 0.3	22.2 ± 0.6	2.6 ± 1.2	4.46	9.10	6.61	11.51
Ti_22_Zr_15_Hf_41_Cr_22_	21.7 ± 0.3	14.1 ± 0.2	40.4 ± 0.4	21.6 ± 0.3	2.2 ± 0.2	4.44	8.87	6.19	10.91
Ti_21_Zr_16_Hf_25_Cr_38_	20.5 ± 0.1	16.0 ± 0.1	25.2 ± 0.0	38.3 ± 0.2	0.0 ± 0.0	4.77	10.41	8.61	11.08

^1^ VEC: valence electron concentration. ^2^ δ: atomic-size difference. ^3^ Δ*H*_mix_: mixing enthalpy. ^4^ Δ*S*_mix_: mixing entropy.

**Table 3:** Mechanical properties and residual stresses of TiZrHf(Ta, Y, Cr) films.

Sample	*H* ^1^ (GPa)	*E* ^2^ (GPa)	*W_e_* ^3^ (%)	*σ* ^4^ (GPa)
Ti_31_Zr_33_Hf_36_	7.4 ± 0.8	179 ± 14	31	−0.13 ± 0.00
Ti_24_Zr_23_Hf_27_Ta_26_	4.7 ± 0.6	104 ± 9	32	−0.23 ± 0.00
Ti_30_Zr_20_Hf_25_Ta_25_	4.6 ± 0.3	100 ± 4	28	0.00 ± 0.00
Ti_21_Zr_30_Hf_25_Ta_24_	5.0 ± 0.3	101 ± 3	33	−0.14 ± 0.24
Ti_20_Zr_18_Hf_38_Ta_24_	5.1 ± 0.5	105 ± 6	30	−0.04 ± 0.11
Ti_17_Zr_17_Hf_26_Ta_40_	5.4 ± 0.3	114 ± 3	30	−0.14 ± 0.05
Ti_24_Zr_23_Hf_27_Y_26_	6.0 ± 0.6	106 ± 6	33	−0.48 ± 0.02
Ti_29_Zr_21_Hf_26_Y_24_	7.4 ± 0.5	114 ± 6	37	−0.54 ± 0.01
Ti_22_Zr_30_Hf_27_Y_21_	7.9 ± 0.5	131 ± 7	36	−0.41 ± 0.13
Ti_21_Zr_19_Hf_42_Y_18_	7.9 ± 0.5	134 ± 9	38	−0.46 ± 0.07
Ti_19_Zr_18_Hf_26_Y_37_	6.1 ± 0.3	106 ± 4	37	−0.74 ± 0.04
Ti_29_Zr_19_Hf_26_Cr_26_	6.6 ± 0.1	105 ± 3	45	−0.24 ± 0.00
Ti_31_Zr_17_Hf_28_Cr_24_	6.8 ± 0.0	104 ± 1	44	−0.27 ± 0.01
Ti_24_Zr_26_Hf_27_Cr_23_	5.3 ± 0.4	91 ± 5	44	−0.15 ± 0.00
Ti_22_Zr_15_Hf_41_Cr_22_	6.5 ± 0.0	105 ± 1	43	−0.43 ± 0.01
Ti_21_Zr_16_Hf_25_Cr_38_	8.4 ± 0.1	113 ± 1	48	−0.28 ± 0.01

^1^ *H*: hardness. ^2^ *E*: elastic modulus. ^3^ *W_e_*: elastic recovery. ^4^ *σ*: residual stress.

**Table 4:** Corrosive characteristics of the bare SUS420 substrate and TiZrHf(Ta, Y, Cr) films.

Sample	*E_corr_* ^1^ (mV)	*I_corr_* ^2^ (μA/cm^2^)	*R_p_* ^3^ (Ω·cm^2^)	*β_a_* ^4^ (mV)	*β*c ^5^ (mV)	*R_p_* Ratio
SUS420	−345	4.826	7.7 × 10^3^	132.6	238.5	1.0
Ti_31_Zr_33_Hf_36_	−354	0.338	1.9 × 10^5^	472.3	221.1	25.2
Ti_24_Zr_23_Hf_27_Ta_26_	−714	2.129	2.4 × 10^4^	169.4	360.1	3.1
Ti_30_Zr_20_Hf_25_Ta_25_	−281	1.497	1.7 × 10^4^	66.3	443.2	2.2
Ti_21_Zr_30_Hf_25_Ta_24_	−388	1.330	3.4 × 10^4^	139.6	396.2	4.4
Ti_20_Zr_18_Hf_38_Ta_24_	−199	0.231	1.0 × 10^5^	65.5	366.7	13.6
Ti_17_Zr_17_Hf_26_Ta_40_	−251	0.221	1.2 × 10^5^	88.4	178.2	15.1
Ti_24_Zr_23_Hf_27_Y_26_	−619	6.291	2.9 × 10^3^	47.4	318.8	0.4
Ti_29_Zr_21_Hf_26_Y_24_	−372	0.418	6.9 × 10^4^	95.3	216.9	9.0
Ti_22_Zr_30_Hf_27_Y_21_	−578	18.120	3.1 × 10^3^	178.2	470.9	0.4
Ti_21_Zr_19_Hf_42_Y_18_	−199	15.414	1.2 × 10^3^	46.1	473.2	0.2
Ti_19_Zr_18_Hf_26_Y_37_	−997	0.551	8.3 × 10^4^	254.3	179.2	10.8
Ti_29_Zr_19_Hf_26_Cr_26_	−225	0.269	2.0 × 10^5^	359.3	190.1	26.2
Ti_31_Zr_17_Hf_28_Cr_24_	−202	0.064	4.5 × 10^5^	101.9	184.8	58.1
Ti_24_Zr_26_Hf_27_Cr_23_	−180	0.140	3.5 × 10^5^	301.6	176.7	45.1
Ti_22_Zr_15_Hf_41_Cr_22_	−243	0.319	7.8 × 10^4^	78.3	215.1	10.2
Ti_21_Zr_16_Hf_25_Cr_38_	−201	0.133	3.3 × 10^5^	318.5	146.6	42.7

^1^ *E_corr_*: corrosion potential. ^2^ *I_corr_*: corrosion current density. ^3^ *R_P_*: polarization resistance. ^4^ *β_a_*: anodic Tafel slope. ^5^ *β_c_*: cathodic Tafel slope.

**Table 5:** Mechanical properties of as-deposited and annealed TiZrHf(Ta, Y, Cr) films.

Sample	Hardness (GPa)			Elastic Modulus (GPa)		
	RT	500 °C	700 °C	RT	500 °C	700 °C
Ti_31_Zr_33_Hf_36_	7.4 ± 0.8	11.6 ± 0.2	18.8 ± 1.7	104 ± 9	171 ± 2	241 ± 14
Ti_24_Zr_23_Hf_27_Ta_26_	4.7 ± 0.6	7.9 ± 0.6	12.5 ± 0.4	100 ± 4	138 ± 6	182 ± 3
Ti_30_Zr_20_Hf_25_Ta_25_	4.6 ± 0.3	7.4 ± 0.2	11.4 ± 1.2	101 ± 3	131 ± 3	196 ± 13
Ti_21_Zr_30_Hf_25_Ta_24_	5.0 ± 0.3	6.9 ± 0.3	12.2 ± 0.5	105 ± 6	128 ± 5	193 ± 4
Ti_20_Zr_18_Hf_38_Ta_24_	5.1 ± 0.5	6.8 ± 0.6	11.2 ± 0.4	114 ± 3	135 ± 8	188 ± 5
Ti_17_Zr_17_Hf_26_Ta_40_	5.4 ± 0.3	7.5 ± 0.3	12.1 ± 0.8	106 ± 6	144 ± 5	192 ± 7
Ti_24_Zr_23_Hf_27_Y_26_	6.0 ± 0.6	9.1 ± 0.5	10.4 ± 0.6	114 ± 6	139 ± 3	184 ± 9
Ti_29_Zr_21_Hf_26_Y_24_	7.4 ± 0.5	8.4 ± 0.4	10.8 ± 0.5	131 ± 7	141 ± 7	189 ± 7
Ti_22_Zr_30_Hf_27_Y_21_	7.9 ± 0.5	8.7 ± 0.3	9.3 ± 0.4	134 ± 9	132 ± 3	172 ± 4
Ti_21_Zr_19_Hf_42_Y_18_	7.9 ± 0.5	11.6 ± 0.4	8.5 ± 0.4	106 ± 4	199 ± 2	170 ± 4
Ti_19_Zr_18_Hf_26_Y_37_	6.1 ± 0.3	9.8 ± 0.7	15.8 ± 1.1	104 ± 9	165 ± 6	236 ± 10
Ti_29_Zr_19_Hf_26_Cr_26_	6.6 ± 0.1	10.5 ± 0.6	16.4 ± 3.1	105 ± 3	142 ± 6	202 ± 23
Ti_31_Zr_17_Hf_28_Cr_24_	6.8 ± 0.0	11.1 ± 0.2	20.6 ± 0.6	104 ± 1	152 ± 2	241 ± 6
Ti_24_Zr_26_Hf_27_Cr_23_	5.3 ± 0.4	9.0 ± 0.7	15.0 ± 5.2	91 ± 5	139 ± 10	205 ± 43
Ti_22_Zr_15_Hf_41_Cr_22_	6.5 ± 0.0	11.3 ± 0.4	19.0 ± 0.4	105 ± 1	154 ± 2	223 ± 3
Ti_21_Zr_16_Hf_25_Cr_38_	8.4 ± 0.1	11.6 ± 0.1	19.5 ± 0.2	113 ± 1	154 ± 1	234 ± 2

## Data Availability

The raw data supporting the conclusions of this article will be made available by the authors on request.
